# Different Aspects of Emotional Awareness in Relation to Motor Cognition and Autism Traits

**DOI:** 10.3389/fpsyg.2019.02439

**Published:** 2019-10-30

**Authors:** Charlotte F. Huggins, Isobel M. Cameron, Justin H. G. Williams

**Affiliations:** ^1^Translational Neuroscience, Institute of Medical Science, School of Medicine, Medical Sciences and Nutrition, University of Aberdeen, Aberdeen, United Kingdom; ^2^Medical Education, Institute of Applied Health Sciences, School of Medicine, Medical Sciences and Nutrition, University of Aberdeen, Aberdeen, United Kingdom

**Keywords:** emotional awareness, motor cognition, motor empathy, emotion differentiation, autistic traits, emotional granularity

## Abstract

Emotion is inherently embodied, formulated through bodily sensation, as well as expressed and regulated through action. Both expressing one’s own emotions and understanding the emotional actions of others are common areas of difficulty in autism. Moreover, reduced emotional awareness is also thought to be problematic in autism, and such difficulties may be mediated by impaired motor cognition. We aimed to examine how intensity of emotional experience and ability to differentiate between one’s own emotions relates to motor empathy and autistic traits. We hypothesized that greater motor cognition would be associated with greater emotional intensity and more refined emotion differentiation. Participants from the general population (*N* = 160) completed the Actions and Feelings Questionnaire (AFQ), a self-report measure assessing motor cognition, alongside the Broad Autism Phenotype Questionnaire and an emotion elicitation task. Motor cognition was significantly associated with more intense emotional experiences but not with ability to differentiate between similar emotions. Autistic traits, particularly social aloofness, predicted less emotion differentiation and lower scores on the animation subscale of the AFQ. We suggest that whereas as intensity of experience may be dependent on sensorimotor representation of emotions, differentiation requires additional cognitive functions such as language understanding. A dissociation between awareness of intensity and differentiation may be critical for understanding emotional difficulties in autism.

## Introduction

The relationship between action and emotion is central to theories of embodied cognition. Embodied cognition represents a general school of thought characterized by many theories, though best summarized with the general idea that the mind is rooted in the whole body, rather than being a piece of “software” installed in the “hardware” of the brain (Marmeleira and Santos, 2019). Within embodiment theories, interaction between the body and the environment drives the formation of perception, cognitions, and knowledge (Varela et al., 2017), as well as emotion (Niedenthal et al., 2005; Damasio, 2018).

On a practical level, emotion is almost always accompanied by action, whether intentional (such as making planned movements to address the cause of an emotion) or unintentional (such as in a reactive facial expression). These actions serve to both regulate emotional experiences and communicate them to others. From a commonsense perspective, removing action from emotion eliminates much of the real-world context in which they are experienced. As such, a holistic science of emotion should also examine the role of motor action.

On a theoretical level, it has been suggested that emotional experiences evolved in order to motivate action (Damasio, 2018), from both simple actions (such as seeking shelter to alleviate discomfort) to more complex, planned actions (such as comforting others due to a sense of sympathy). Actions and bodily experiences are also closely tied to how emotions arise on a moment-to-moment level. Interoception, awareness of one’s own bodily sensations has long been considered fundamental to how emotion arises (James, 1884; Damasio, 1994; Craig, 2003; Critchley et al., 2004; Seth, 2013), and contemporary constructionist accounts argue that emotion is constructed from interoceptive sensation (Barrett, 2017; Hoemann et al., 2019).

Despite the growing body of work demonstrating the importance of bodily awareness to emotion, how action awareness relates to emotion is not a frequent topic of discussion. Yet empirical findings indicate that action influences how emotions are experienced. Strack et al. (1988) found that participants asked to hold a pen between their teeth, stimulating the muscles involved in smiling, rated cartoons as more amusing. Similar effects have been found for other facial actions and ratings of emotions such as disgust (Lewis, 2012) and ratings of neutral stimuli (Mori and Mori, 2013) and others’ emotional states (Hyniewska and Sato, 2015). Moreover, patients who undergo cosmetic botulinum toxin injections, limiting the movement of their face, report diminished emotional intensity post-treatment (Davis et al., 2010). This illustrates how one’s own bodily sensations and actions influence one’s own subjective experiences of emotion and judgments of others’ emotional states.

These facial and bodily feedback effects may be central to how we understand the emotions of others. Mimicry, the unintentional copying of others’ facial and bodily actions may form the earliest mechanism by which emotional labels and expressions are learned and understood. Allen and Heaton (2010) suggest that infants learn facial expressions corresponding to their own emotions by observing the caregiver’s mimicked facial expression. To give an example, an infant who feels happy and subsequently smiles will likely observe a mimicked smile on their caregiver’s face. This allows the infant to learn the connection between the experienced emotion and how this is expressed through action and language. In later life, these perception-action links may facilitate emotional contagion (Niedenthal, 2007; Decety and Meyer, 2008; Bird and Viding, 2014), whereby just viewing the facial expressions of others, without mimicry, simulates the accompanying emotional state in the self. This emotional contagion mechanism forms the basis of more complex empathy (Van der Graaff et al., 2016), allowing individuals to quickly identify and understand emotional states in others and respond to them appropriately.

An important step in this process is for individuals to identify their own emotional states and distinguish these clearly from the emotions of others. While the ability to recognize the emotions of others has long been a frequent topic of study in autism research, the role of awareness of one’s own emotions has largely been historically overlooked. Over the past decade, there has been a renewed interest in emotional awareness within mental health research. There is tremendous natural variability in how individuals identify and report their own emotional states (Barrett and Satpute, 2019). Such differences can be seen both in terms of how strongly emotions are experienced, as well as how finely individuals differentiate between their own emotional experiences (Barrett, 2009). For instance, while some experience emotions as intense clearly differentiated states (e.g., discriminating feeling ‘excited’ from feeling ‘hopeful’), others struggle to make these finer distinctions, experiencing emotion as generally “good” or “bad.” This ability to differentiate between one’s own emotions is thought to be a key predictor of emotional regulation (Barrett et al., 2001), as well as predicting socioemotional outcomes in a wide range of psychiatric disorders (Smidt and Suvak, 2015), including autism.

Within autism research, emotional awareness is largely measured through alexithymia. Alexithymia is a construct originally derived from work with psychosomatic patients (Sifneos, 1973), describing a common pattern of impoverished imagination, utilitarian thinking, and communication difficulties, particularly a dry and literal manner of speech. Such patients were noted to have difficulty identifying and describing their own emotional experiences, even when prompted to do so by therapists. In contemporary research, alexithymia is frequently defined as difficulties identifying and describing emotions (e.g., Bird and Cook, 2013). The original behavioral aspects (such as utilitarian thinking and literal speech) of alexithymia are often not discussed in contemporary research, although cognitive and affective subtypes of alexithymia have been proposed (Bermond et al., 2010).

Most work on alexithymia in autism has relied upon self-report questionnaires, most prominently the Toronto Alexithymia Scale (TAS-20; Bagby et al., 1994). Autistic people tend to score more highly on these outcomes compared to non-autistic people (Hill et al., 2004; Berthoz and Hill, 2005), indicating diminished emotional awareness. Recent meta-analytic work has confirmed these differences are consistent across studies using the TAS-20 (Kinnaird et al., 2019). Moreover, Bird and Cook (2013) proposed that elevated alexithymia predicts greater difficulties with recognizing the emotions of others and diminished emotional contagion observed in autistic populations. Moreover, the contemporary definition of alexithymia has obvious conceptual similarities to emotional awareness and may be considered to represent the “lower end” of emotional awareness abilities (Barrett, 2006). Alexithymia is also strongly connected to interoception (Brewer et al., 2016). Accumulating evidence suggests that greater interoceptive accuracy predicts lower alexithymia and thus greater awareness of one’s own emotions (Murphy et al., 2018), and this association may have significant implications for the etiology and treatment of mental health problems (Murphy et al., 2017).

The action-perception-emotion links outlined above may be particularly important in autism. It has long been noted that autistic children are less likely to imitate others compared to their neurotypical peers, and it is suggested that these early difficulties slow the development of social and emotional skills, causing cascading effects across the lifespan (Rogers and Pennington, 1991; Williams et al., 2001). Moreover, as imitation is suggested to facilitate recognition of others’ facial expressions (Niedenthal, 2007) and establish social rapport (Chartrand and Bargh, 1999), diminished imitation in development may lead to difficulties with emotion recognition and social success in autistic people later in life (Winkielman et al., 2009). Subsequently, motor cognition and empathy remain important issues in both the study of emotion and autism.

Yet compared to cognitive and emotional empathy, motor empathy has received relatively little attention in research. Moreover, few studies have examined motor empathy in conjunction with emotional self-awareness and autism. One potential reason is that measurement of motor empathy is usually challenging, requiring methods such as facial electromyography or visual action coding. Furthermore, motor empathy is often not explicitly measured in self-report measures of empathy (e.g., the Interpersonal Reactivity Index; Davis, 1980). Potentially, the creation of a self-report measure of motor empathy and cognition may allow for more diverse research in this field.

To address this gap in the literature, Williams et al. (2016) developed the Actions and Feelings Questionnaire (AFQ), a self-report questionnaire intended to measure self-awareness of sensorimotor involvement in social and emotional behavior. The AFQ measures awareness of the emotional actions of others, one’s own use of imagery, and how frequently one uses gesture. AFQ scores are significantly lower in autistic than neurotypical adults (Williams and Cameron, 2017), demonstrating potential discriminative validity and aligning with the wider findings of diminished mimicry and imitation in autism.

Moreover, AFQ scores correlate with somatosensory cortex activation during facial imitation tasks (Williams et al., 2016). Based on this, the authors argued that the AFQ assesses self-awareness of emotional actions, and this awareness may predict emotional awareness. There is a commonsense logic to this proposal. If awareness of bodily sensations, as measured through interoception, predicts emotional awareness, it would follow that awareness of bodily actions may similarly facilitate more refined emotional awareness. If that is the case, higher AFQ scores may be associated with less intense and more poorly differentiated experience of one’s own emotional experiences, which would subsequently predict greater difficulty understanding the emotions of others. Such a relationship may be particularly important in understanding the empathic and emotional difficulties seen in autism.

Despite these findings, the relationship of the AFQ to actual emotional experience has yet to be explicitly measured. Our study aims to address this gap by comparing AFQ scores to experimental measures of emotional intensity and differentiation and to autistic traits. Based on the above literature, it is predicted that higher AFQ scores will be associated with more intense and better differentiated emotional experience.

## Methodology

### Ethics

This study was approved by the University of Aberdeen School of Psychology Ethics Committee.

### Participants

One hundred and seventy-one adult participants (31 males and 140 females) were recruited from the Aberdeen area, through undergraduate participants’ pool, mailing lists, community advertisements, social media advertisements, and public engagement. Participants from the University of Aberdeen School of Psychology were offered course credit. All other participants were offered £10 cash to thank them for their time. Eleven participants were excluded from final analyses. Five participants had missing PED-task data due to a technical error, 5 did not complete the AFQ, and 1 had significant amounts of missing data.

Following exclusions, there was a final sample of 160 participants (132 females and 28 males), with a median age of 20 (IQR = 3; range = 17–59). Of these 160 participants, 148 were undergraduate students, 3 were post-graduate students, and 9 were non-students.

### Emotional Intensity and Differentiation

Emotional intensity and differentiation were measured with an image-rating paradigm based on Erbas et al.’s (2014) Photo Emotion Differentiation Task (PED task). In the PED task, participants view emotional images paired with different emotion terms, rating how strongly the image evokes each discrete emotional state. While Erbas and colleagues’ original paradigm exclusively used negative images and words, the current PED task also included positive images and terms.

It should be noted that the PED task does not assess how accurately participants identify the emotional expressions of others. Instead, participants are asked to rate *their own* subjective emotional responses to the stimuli. This differentiates the task from popular tasks such the Reading the Mind in the Eyes test (Baron-Cohen et al., 2001), which focuses on ability to identify the emotions of *others*. The fact that participants are expected to rate their own emotional responses, rather than the emotional qualities of the images, is stressed multiple times in both the oral and written instructions.

Emotional stimuli were taken from the Nencki Affective Picture System (NAPS; Marchewka et al., 2014), 10 due to their high negative valence ratings and 10 due to their high positive valence ratings. Emotion terms consisted of five positive (happy, enthusiastic, amused, hopeful, and relaxed) and five negative (ashamed, nervous, angry, sad, and guilty) terms.

Each of the 20 images was presented with each of the 10 emotion terms once, resulting in a total of 200 unique ratings. In each case, participants had to rate how intensely that particular image made them feel the given emotion term, on a 0–6 Likert scale. A score of 0 indicated that they did not feel the target emotion in response to the image at all, and 6 indicate that they felt the target emotion very strongly.

**Figure 1:**
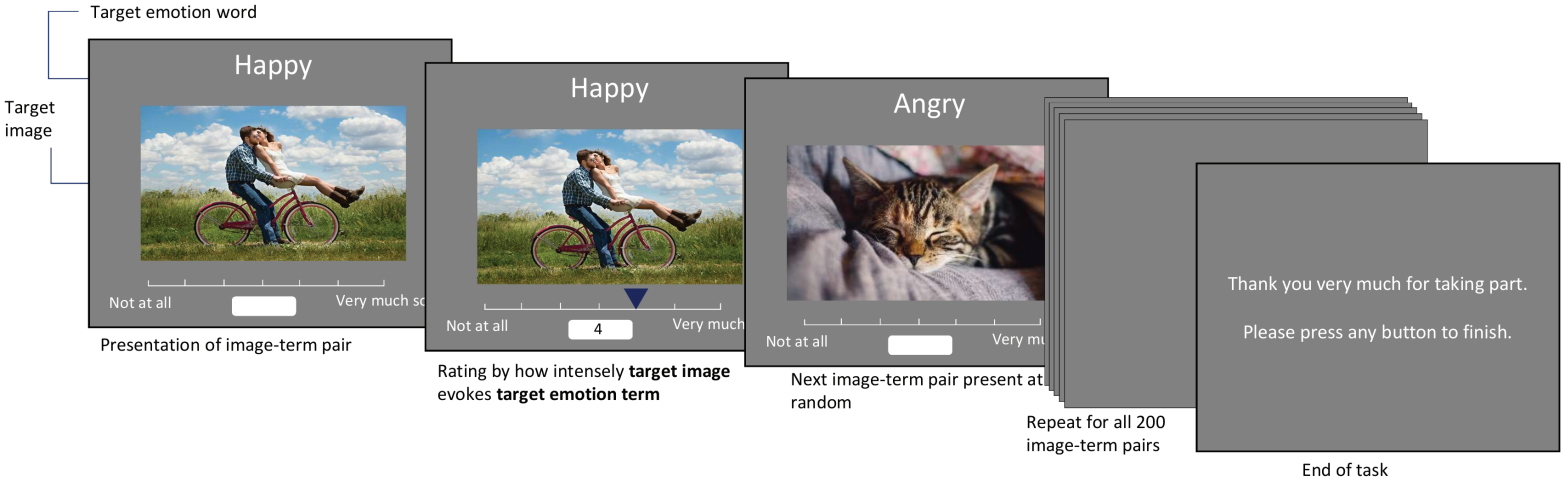
Outline of PED-task procedure.

Participants first completed two practice items alongside the experimenter. The experimenter then ensured participants understood the task, particularly that they were expected to rate their own emotional experiences, rather than any inherent qualities of the image itself. After that, participants completed the experimental trials alone. Each image-term pairing was presented one at a time on a computer screen. The order of the image-term pairs was randomized for each participant. Participants could take as long as they wished to rate each item (see Figure 1 for diagram of PED-task procedure).

Indices of emotional intensity and differentiation were calculated from these ratings. Intensity reflects general strength of emotional response, whereas differentiation reflects how well participants discriminate between their own discrete emotional states. Details of how emotional intensity and differentiation were calculated can be seen in the statistical analyses section.

### The Actions and Feelings Questionnaire

Participants completed the Actions and Feelings Questionnaire (Williams et al., 2016), an 18-item self-report measure assessing awareness of action related to emotion. The AFQ assesses how aware individuals are of their own and others’ motor actions in social and emotional behavior. The AFQ is significantly correlated with Empathy Quotient (EQ; Baron-Cohen and Wheelwright, 2004) scores (Williams et al., 2016) and shows good discriminant validity between autistic and typical populations (Williams and Cameron, 2017). Items are summed to produce total scores, with higher scores reflecting greater motor empathy.

The AFQ has three subscales: Feelings, Animation, and Imagery. The Feelings subscale reflects sensitivity to others’ emotionally expressive motor actions, assessed through items such as “I tend to pick up on people’s body language.” The Animation subscale reflects tendency to express emotion through body language, facial expression, and tone of voice, through items such as “I get animated when I am enthusiastic in conversation.” Finally, the Imagery subscale reflects how strongly and frequently people rely on action imagery, with items such as “In my mind’s eye, I often see myself doing things.”

### Autistic Traits

Autistic traits were assessed with the Broad Autism Phenotype Questionnaire (BAPQ; Hurley et al., 2007), a 36-item self-report questionnaire assessing the presence of autistic traits in the general population. BAPQ scores are significantly higher in the parents of autistic than non-autistic children (Hurley et al., 2007; Sasson et al., 2013) as well as in autistic than non-autistic adults (Nishiyama et al., 2014). Previous studies suggest that the BAPQ outperforms comparable autistic trait measures in terms of internal consistency, convergent validity, and stability of factor structure (Ingersoll et al., 2011). Finally, the BAPQ was explicitly designed to detect the presence of autistic traits in the general population, rather than to screen for autistic symptoms in a clinical group.

There are three subscales in the BAPQ: Aloofness, Pragmatic language difficulties, and Rigidity. The Aloof subscale reflects a lack of interest in or little enjoyment in casual social interaction, assessed with items like “I like being around other people.” The Pragmatic language subscale refers to difficulties in social language, such as in communicating ideas and holding reciprocal conversation, assessed with items such as “I find it hard to get my words out smoothly.” Finally, Rigidity reflects difficulty adjusting to change and preference for routine, assessed with items such as “I am comfortable with unexpected changes in plans.” Scores are averaged to produce totals, with higher scores reflecting greater autistic traits.

### Procedure

The current dataset is pooled from two different studies. In each study, the AFQ, BAPQ, and PED-task procedures were identical.

In the first study, participants also took part in two tasks not reported in the current study: a facial imitation task and an image judgment task. In the second study, participants took part in a different image judgment task alongside a working memory task.

In both cases, participants took part in a quiet, private university room. The experimenter orally explained the study to the participant, taking informed consent after answering any questions and ensuring they understood the study.

During the first study, participants took part in the PED task first, followed by the other tasks, and finally the self-report questionnaires. By contrast, participants in the second study took part in the image judgment task and working memory task first, followed by the PED task and finally the self-report questionnaires.

### Statistical Analyses

PED-task data were used to calculate two outcomes of emotional self-awareness: emotional intensity (how intensely individuals responded to affective stimuli) and emotion differentiation (how well participants differentiated between discrete emotional states).

Emotional intensity was calculated through the mean emotion ratings for congruent image-word pairings (e.g., positive words with positive images). Average intensity was calculated with the mean of positive and negative intensity. These scores represented general strength of emotional response, with higher scores reflecting greater emotional intensity.

Emotion differentiation was calculated through intra-class correlations (ICCs) of congruent image-word pairings, in line with previous work (Erbas et al., 2014, 2019). ICCs reflect strength of correlation between a participant’s ratings of the same emotional stimuli by different emotional terms. Higher correlations reflect application of emotion terms of the same valence to the same stimuli in similar ways, reflecting poorer differentiation between discrete emotional states.

Two-way random effects, multiple-rater ICCs with consistency (also known as Cronbach’s alpha) were used to calculate all differentiation scores (see Koo and Li, 2016, for full details on different types of ICCs). These were calculated for ratings of images across all emotion terms of the same valence (e.g., ratings of all positive images by all positive emotion terms).

Some differentiation scores fell below zero – 20 positive (11.9%) and 12 negative (7.5%) scores, in line with previous findings (Erbas et al., 2019). As ICCs should theoretically only range between 0 and 1 (Shrout and Fleiss, 1979), it is unlikely these scores can be meaningfully interpreted. In line with previous research using this method (Erbas et al., 2019), these scores were excluded from further emotion differentiation analyses and remaining scores were transformed through Fischer’s *r*-to-*z*. Higher scores reflect stronger correlations between ratings of discrete emotional states and thus poorer emotion differentiation.

## Results

All scores were normally distributed (see Table 1 for averages, including averages by gender).

**Table 1:** Average and variances for all outcomes.

	Full sample	Males	Females
	Mean (SD)	Mean (SD)	Mean (SD)
**AFQ total**	36.64 (6.40)	34.54 (6.15)	37.09 (6.39)
1. Feelings	18.99 (3.86)	18.82 (3.21)	19.03 (4.00)
2. Animation	10.79 (2.58)	9.54 (2.98)	11.05 (2.41)
3. Imagery	6.86 (2.42)	6.18 (2.21)	7.01 (2.44)
**BAPQ total**	2.75 (0.55)	2.81 (0.53)	2.74 (0.55)
1. Aloof	2.66 (0.77)	2.79 (0.61)	2.63 (0.80)
2. Pragmatic	2.69 (0.63)	2.76 (0.65)	2.67 (0.63)
3. Rigidity	2.91 (0.76)	2.88 (0.77)	2.91 (0.76)
**Average intensity**	3.16 (0.97)	2.94 (0.75)	3.21 (1.00)
1. Negative intensity	3.03 (1.05)	2.51 (0.80)	3.14 (1.07)
2. Positive intensity	3.29 (1.15)	3.36 (0.96)	3.28 (1.19)
**Average differentiation**	0.81 (0.28)	0.82 (0.28)	0.80 (0.28)
1. Negative differentiation	0.78 (0.36)	0.75 (0.34)	0.78 (0.36)
2. Positive differentiation	0.89 (0.39)	0.92 (0.42)	0.89 (0.38)

*AFQ, Actions and Feelings Questionnaire; BAPQ, Broad Autism Phenotype Questionnaire. Differentiation scores have all underwent Fischer’s r-to-z transformation*.

Due to the higher proportion of female than male participants, one-way ANOVAs were conducted to examine gender differences in self-report and PED-task scores. No gender differences emerged in total AFQ scores [*F*(1, 158) = 3.741, *p* = 0.055]. No gender differences emerged in BAPQ scores [*F*(1, 158) = 0.420, *p* = 0.518].

Comparing PED-task outcomes, no gender differences emerged in positive intensity [*F*(1, 158) = 0.126, *p* = 0.724]. A significant difference emerged in negative intensity [*F*(1, 158) = 8.613, *p* = 0.004], with female participants (*M* = 3.14, SD = 1.07) having more intense negative emotional responses to stimuli than male participants (*M* = 2.51, SD = 0.80). No gender differences emerged in emotion differentiation [positive differentiation: *F*(1, 138) = 0.121, *p* = 0.728; negative differentiation: *F*(1, 146) = 0.290, *p* = 0.591].

As gender differences were largely non-significant, gender was not controlled for in any further statistical analyses.

### Broad Autism Phenotype Questionnaire and Actions and Feelings Questionnaire

**Figure 2:**
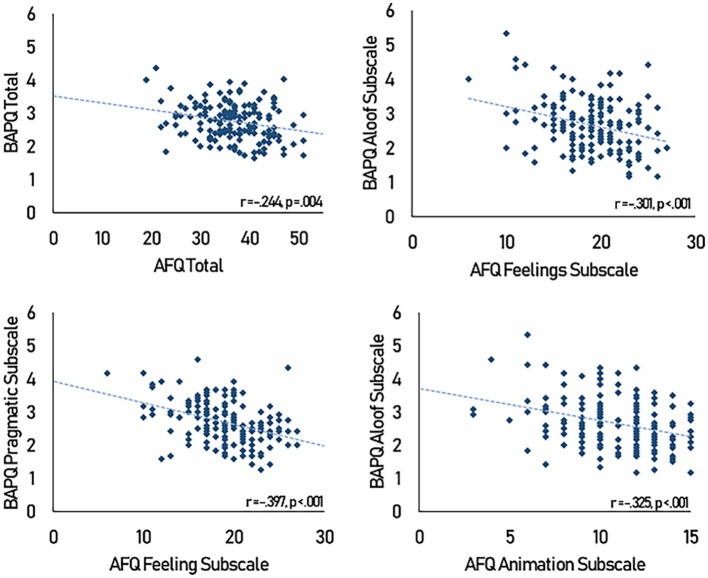
Scatterplot of AFQ scores against BAPQ scores.

Total BAPQ scores were significantly correlated with total AFQ scores [*r*(158) = −0.244, 95% CI (−0.384, −0.092), *p* = 0.004]. To examine this relationship in greater detail, Pearson’s correlations between the subscales of the AFQ and BAPQ were conducted. To control for multiple comparison effects and Type I errors, Bonferroni’s multiple comparison correction was used, and minimum *p* value for statistical significance was subsequently set to 0.005. By these standards, AFQ Feelings was significantly correlated with BAPQ Aloof [*r*(158) = −0.301, 95% CI (−0.436, −0.153), *p* < 0.001] and BAPQ Pragmatic [*r*(158) = −0.397, 95% CI (−0.520, −0.257), *p* < 0.001]. AFQ Animation was also significantly correlated with BAPQ Aloof [*r*(158) = −0.325, 95% CI (−0.457, −0.179), *p* < 0.001] (see Figure 2 for scatterplots).

### Photo Emotion Differentiation-Task Outcomes

Positive and negative intensity were well correlated [*r*(158) = 0.548, 95% CI (0.426, 0.649), *p* < 0.001], indicating that scores reflect general intensity of emotional response rather than positive or negative bias.

Positive and negative differentiation weakly correlated [*r*(128) = 0.196, 9% CI (0.005, 0.364), *p* = 0.026], indicating that participants tended to perform similarly when asked to differentiate between positive and negative emotions. A paired-sample *t* test found a significant difference between positive and negative differentiation [*t*(129) = 2.844, *p* = 0.005], indicating that participants tended to differentiate more between negative than positive emotional terms.

To confirm differentiation was not simply an artifact of general emotional intensity, linear regressions were conducted to assess how intensity predicted differentiation. Negative emotional intensity did not significantly predict negative differentiation [*F*(1, 146) = 3.568, *p* = 0.061]. Similarly, positive intensity did not predict positive differentiation [*F*(1, 138) = 0.393, *p* = 0.532]. These findings indicate that differentiation is unlikely to be an artifact of general emotional intensity.

### Actions and Feelings Questionnaire and Photo Emotion Differentiation-Task Outcomes

**Figure 3:**
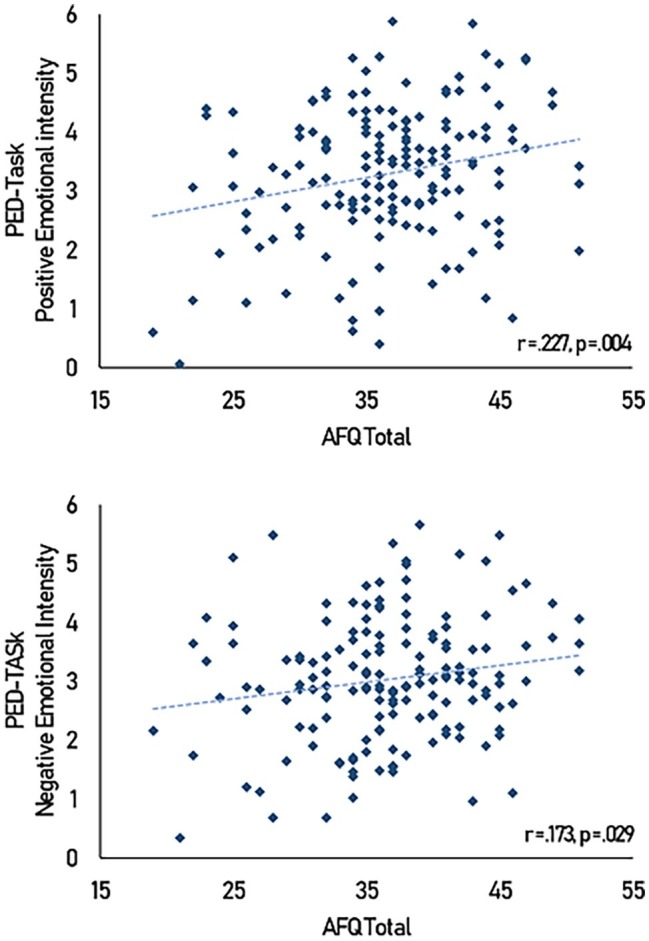
Scatterplot of AFQ scores against emotional intensity scores.

Total AFQ scores were correlated with both positive [*r*(158) = 0.227, 95% CI (0.075, 0.369), *p* = 0.004] and negative [*r*(158) = 0.173, 95% CI (0.018, 0.319), *p* = 0.029] emotional intensity. These associations indicate that motor empathy is associated with more intense responses to both positive and negative emotional stimuli (see Figure 3).

Step-wise linear regressions were conducted to examine which AFQ subscales predicted positive and negative emotional intensity, respectively. AFQ Animation scores (*β* = 0.181, *p* = 0.022) significantly predicted positive emotional intensity [*F*(1,158) = 5.379, *p* = 0.022, *R*^2^ = 0.033]. Neither Feelings (*β* = 0.105, *p* = 0.202) nor Imagery (*β* = 0.118, *p* = 0.153) significantly predicted positive emotional intensity.

AFQ Imagery scores (*β* = 0.221, *p* = 0.005) significantly predicted greater negative emotional intensity [*F*(1,158) = 8.092, *p* = 0.002, *R*^2^ = 0.043]. Neither Feelings (*β* = 0.027, *p* = 0.733) nor Animation (*β* = 0.063, *p* = 0.763) significantly predicted negative emotional intensity.

Total AFQ scores were not significantly associated with positive differentiation (*r* = −0.048, *p* = 0.570) or negative differentiation (*r* = 0.001, *p* = 0.990).

### Broad Autism Phenotype Questionnaire and Photo Emotion Differentiation-Task Outcomes

Total BAPQ scores were not significantly associated with negative emotional intensity scores [*r*(158) = −0.014, 95% CI (−0.169, 0.141), *p* = 0.859], but a weak correlation did emerge for positive intensity scores [*r*(158) = −0.175, 95% CI (−0.322, −0.021), *p* = 0.027]. These findings suggest that greater autistic traits were only associated with diminished positive emotional responses to positive stimuli.

Step-wise linear regressions were used to examine how BAPQ subscales predicted positive emotional intensity. The aloof subscale of the BAPQ (*β* = −0.340, *p* < 0.001) significantly predicted lower positive intensity [*F*(1, 158) = 13.565, *p* < 0.001, *R*^2^ = 0.079]. Pragmatic language difficulties (*β* = 0.018, *p* = 0.832) and rigidity (*β* = 0.133, *p* = 0.117) were not significant and subsequently excluded from the model.

Total BAPQ scores correlated with average differentiation [*r*(128) = 0.238, 95% CI (0.069, 0.394), *p* = 0.006], indicating that greater autistic traits were associated with poorer differentiation of emotional states. This relationship was found between BAPQ and positive [*r*(138) = 0.169, 95% CI (−0.027, 0.329), *p* = 0.046] and negative differentiation [*r*(146) = 0.185, 95% CI (0.031, 0.329), *p* = 0.025].

**Figure 4:**
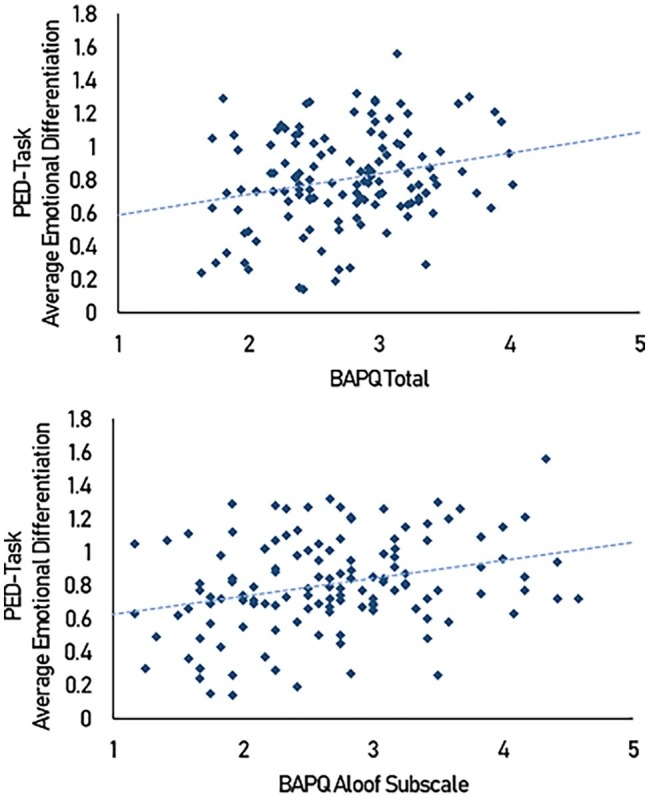
Scatterplot of average emotion differentiation scores against BAPQ scores.

Step-wise linear regressions were used to examine how BAPQ subscales predicted differentiation. Of the three subscales of the BAPQ, only the Aloof subscale (*β* = 0.301, *p* < 0.001) significantly predicted average differentiation [*F*(1, 128) = 12.787, *p* < 0.001, *R*^2^ = 0.087] (see Figure 4). Neither Pragmatic language difficulties (*β* = 0.059, *p* = 0.655) nor Rigidity (*β* = −0.053, *p* = 0.580) reached statistical significance and were subsequently excluded from the model.

**Figure 5:**
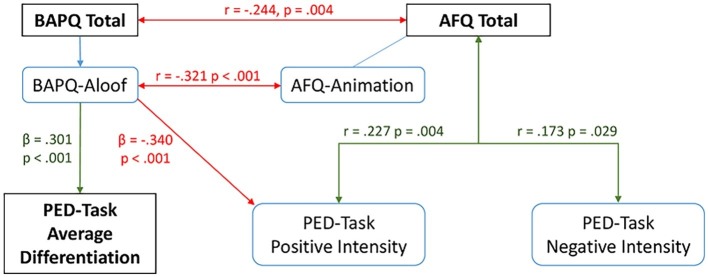
Diagram showing relationships between main variables of interest.

A summary of the relationships between our main outcomes can be seen in Figure 5. AFQ relates to emotional intensity and BAPQ but not to differentiation, whereas higher BAPQ scores predict poorer differentiation and are weakly associated with lower positive emotional intensity.

## Discussion

We examined how motor cognition and empathy, as measured by the AFQ, related to emotional intensity and differentiation in relation to autistic traits. The AFQ was designed to assess awareness of somatomotor representations of actions during social and emotional experiences and was therefore hypothesized to provide a measure of emotional self-awareness (Williams et al., 2016; Williams and Cameron, 2017). Subsequently, it should follow that greater AFQ scores should predict emotional intensity and differentiation. We found only partial support for this hypothesis. Greater scores on the AFQ were associated with more intense emotional responses to stimuli, supporting the notion that greater action-awareness is associated with more intense emotional experiences. However, AFQ was unrelated to ability to differentiate between similar emotional experiences. Instead, differentiation was associated with autistic traits, with greater social aloofness predicting less refined differentiation between one’s own emotional states.

Our findings first indicate that emotional intensity and differentiation can be clearly distinguished from one another. Previous findings suggest that intensity and differentiation scores tend to correlate (Erbas et al., 2019), indicating that individuals who experience more intense emotional responses also tend to differentiate less between similar emotional states. This has raised concerns that differentiation scores may be an artifact of – or confounded by – general emotional intensity. Yet, our findings showed no association between emotional differentiation and intensity. Moreover, differentiation and intensity were associated with different psychosocial outcomes.

Action awareness predicted more intense responses to emotional stimuli. This falls in line with previous studies indicating that bodily and facial action intensifies emotional experience (Strack et al., 1988; Davis et al., 2010; Lewis, 2012). Action awareness was not associated with ability to differentiate between one’s own emotional states. Instead, greater differentiation was associated with lower autistic traits, specifically the Aloofness subscale of the BAPQ. Aloofness scores reflect greater degree of disinterest in social interaction (Hurley et al., 2007), and its association with differentiation may reflect the role of socialization in the formation of emotion concepts.

Contemporary constructionist accounts of emotion argue that discrete categories of emotion do not arise as natural and biological responses, but in fact socially and culturally learned categories are imposed upon our internal somatosensory experiences (Barrett, 2017). These categories of emotion are learned from infancy through socialization (Barrett, 2006; Hoemann et al., 2019), and greater exposure to emotion-related socialization predicts greater emotional awareness (Warren and Stifter, 2008). Much like with other types of learning, this emotional learning process is likely to continue throughout the lifespan. Those with greater social aloofness may be less likely to socialize with others, have fewer opportunities for emotional learning, and thus show less refined use of emotion concepts.

The role of language and verbal IQ cannot be neglected in this discussion. Emotional language forms a key part of how emotion concepts are formed (Barrett, 2006; Hoemann et al., 2019). Subsequently, verbal IQ and language skill may be a key component of individual’s ability to communicate effectively about emotion. Supporting this idea, verbal mental age, not diagnostic status, predicted ability to detect emotions in music in autistic and non-autistic children (Heaton et al., 2008). Similarly, greater verbal ability significantly predicts ability to detect emotions in facial expressions (Montebarocci et al., 2011; Fink et al., 2014). Yet, these findings only reflect an individual’s ability to detect emotional content in external stimuli, not ability to identify one’s own emotional experiences.

Of greater relevance to the current study are findings from the alexithymia literature. Greater self-reported alexithymia is associated with lower verbal IQ (Montebarocci et al., 2011), as well as with impaired language skill and damage to language-related brain regions (Hobson et al., 2018). Alexithymia is also associated with poorer semantic knowledge of emotion terms and less varied use of emotion terms (Wotschack and Klann-Delius, 2013). It must be noted that this work significantly differs from the current study, as these are based on self-reports of alexithymia, rather than differentiation specifically. While little work has directly examined how verbal IQ and language relate to behavioral indices of differentiation, research does suggest that greater verbal IQ predicts more refined differentiation (Israelashvili et al., 2019). As such, while intensity may relate to somatosensory awareness, differentiation may instead draw upon the higher cognitive processes, such as language.

The distinction between intensity and differentiation observed here also bears a resemblance to the proposed distinction between affective and cognitive alexithymia found in the wider literature. While much research focuses on the cognitive aspects of alexithymia, such as difficulty identifying feelings, other researchers have suggested that there is also an affective component of alexithymia (Vorst and Bermond, 2001). Moreover, this affective component of alexithymia is largely neglected in the TAS-20. While cognitive alexithymia is characterized by deficiencies in the ability to identify, verbalize, and analyze emotions, affective alexithymia is associated with diminished emotional arousal and emotional fantasizing (Bermond et al., 2003). As such, there may be subtypes of alexithymia – one characterized by both diminished emotion and accompanying cognition, and one wherein intensity of emotional experience remains intact, but cognition is impaired. These affective and cognitive components of alexithymia show clear similarities to the intensity and differentiation outcomes examined in our current study. Moreover, like our current findings, cognitive and affective alexithymia are suggested to be independent. As such, an individual could have high emotional arousal but diminished ability to identify and articulate these emotions. Neuroscientific findings support this view, finding that cognitive and affective alexithymia are associated with different brain structures (Goerlich, 2018), indicating they are dissociable on a neural level.

While there is limited work examining cognitive and affective alexithymia in autism, existing research suggests that autistic participants have elevated cognitive alexithymia but no differences in affective alexithymia, compared to typical controls (Berthoz and Hill, 2005; Ketelaars et al., 2016; Dijkhuis et al., 2017; Ziermans et al., 2019). This suggests that while autistic people frequently have difficulties with analyzing and describing their emotional experiences, there are no differences in the intensity of experience. This aligns with our finding that greater autistic traits are associated with diminished differentiation but not with lower emotional intensity. As such, our findings may be interpreted as providing support for the cognitive/affective model of alexithymia.

While the cognitive/affective model of alexithymia usefully describes the different ways difficulties in emotional self-awareness may arise, there are some issues with this approach. Large-scale psychometric studies have not found clear evidence of the proposed two-factor structure (Bagby et al., 2009; Preece et al., 2017), leading to the suggestion that the proposed affective components are not a stable part of the alexithymia construct. As such, affective alexithymia and, by extension, emotional intensity may be best understood not as part of alexithymia or emotional awareness *per se*, but as general emotional reactivity. Our findings support this notion, demonstrating that intensity is independent from differentiation, with dissociable psychosocial correlates.

There are a number of limitations to the current study. First, our findings are based on correlational, cross-sectional data. As such, the directionality of these relationships remains unclear, and the long-term implications remain relatively speculative. The correlations found were also relatively weak, but it should be noted that this is typical of studies comparing behavioral and self-reported measures of emotional competence (Brackett and Mayer, 2003; Lumley et al., 2005), and many studies do not find any significant relationship at all. A potentially more pertinent issue is that emotion differentiation abilities fluctuate over time (Erbas et al., 2018), particularly in line with changes in stress. It is therefore necessary to replicate these measures to ensure test-retest validity and control for the impact of psychological distress.

Second, findings are based on autistic traits in a typical population, rather than on clinical samples. Autism is increasingly recognized as a continuum, with autistic traits being dispersed dimensionally across the whole population (Wing, 1988; Constantino and Todd, 2003), making this a useful approach, particularly in terms of recruitment and ethics. Yet, this means that our findings may not generalize to clinical populations. The current study generates hypotheses for testing in populations with autism, particularly in relation to mental health outcomes and emotional regulation.

Finally, verbal IQ and language skill were not directly assessed in our current study. The sample was composed largely of university students and staff, making it likely that verbal IQ was generally high and of limited variability. Regardless, examining emotional and non-emotional language skills represents an important next step in examining how the different aspects of emotional self-awareness relate to motor cognition and autistic traits.

Our findings provide evidence that emotion differentiation is dissociable from general emotional intensity. Moreover, we tentatively propose that while general emotional intensity relates to sensorimotor awareness and tendency, differentiation is related to social learning and may necessitate higher-order cognitive processes. In line with wider studies on autistic samples, autistic traits were associated with poorer differentiation, and this may relate to reduced opportunities for social learning. Distinguishing more clearly between differentiation and intensity in discussions of emotional awareness will allow us to more finely pinpoint where difficulties arise, and how they are best addressed.

## Data Availability Statement

The datasets generated for this study are available on request to the corresponding author.

## Ethics Statement

The studies involving human participants were reviewed and approved by University of Aberdeen School of Psychology Ethics Committee. The patients/participants provided their written informed consent to participate in this study.

## Author Contributions

CH was involved in study design, research and development of materials, data collection, analysis and interpretation, and writing manuscript. JW assisted in study design and development of materials and edited and reviewed the manuscript. IC assisted in study design and contributed to development of materials.

### Conflict of Interest

The authors declare that the research was conducted in the absence of any commercial or financial relationships that could be construed as a potential conflict of interest.
